# News about VDAC1 in Hypoxia

**DOI:** 10.3389/fonc.2016.00193

**Published:** 2016-08-30

**Authors:** N. M. Mazure

**Affiliations:** ^1^CNRS UMR7284, INSERM U1081, Institute for Research on Cancer and Aging, Nice (IRCAN), University of Nice, Nice, France; ^2^CNRS GDR 3697 Micronit (www.micronit.fr)

**Keywords:** cancer, mitochondria dysfunction, VDAC1, hypoxia-inducible factor 1, resistance to cell death

## Abstract

The voltage-dependent anion channel (VDAC) is the main interface between the cytosol and mitochondria of cells. It plays a crucial role in both mitochondrial metabolism and cell death. The main basic function of this channel is to mediate and gate the flux of small ions, metabolites, and adenosine triphosphate. Changes in its structure, and thus conformation, are expected to affect its activity and modulate the ability of cancer cells to expand. In this review, we describe a novel mechanism by which mitochondria of cells in hypoxia, a low level of oxygen, protects from apoptosis. In hypoxia, some mitochondria become enlarged due to hyperfusion. These mitochondria possess a truncated form of VDAC1 (VDAC1-ΔC), which is linked to the higher metabolic capacity and the greater resistance to cell death of hypoxic cells. However, not all of the VDAC1 protein is truncated, but the amount of the full-length form is diminished compared to the amount in normoxic cells. First, we describe how such a decrease effects cell proliferation, respiration, glycolysis, and other processes. Second, we report on a novel mitochondrial-endolysosomal crosstalk that leads to VDAC1 truncation. By pharmacological targeting of VDAC1-ΔC, the production of energy could be turned off and the sensitivity to cell death restored. This could counteract the favorable microenvironment that gives cancer cells a growth advantage and thereby disrupts the balance between life and death, which is controlled by VDAC1.

## Introduction

Mitochondria have evolved over time to take on a symbiotic relationship within eukaryotic cells to produce adenosine triphosphate (ATP) through activation of the electron transport chain (1). The production of ATP is probably the most important function of mitochondria, together with the regulation of apoptosis. Thereby, they are involved in different processes essential to the maintenance of cellular homeostasis. Modifications in metabolism and the redox status, critical steps in tumor cell transformation and progression, make mitochondria attractive targets for therapeutic treatment. Therefore, any modification to cancer cell metabolism and more specifically to mitochondrial metabolism, by increasing reactive oxygen species (ROS) production, or stimulating mitochondrial permeability transition to induce cell death, could be promising new therapeutic strategies (2).

How do mitochondria behave in hypoxia? Hypoxia is a decrease in the oxygen concentration compared to the normal physiological concentration and is a characteristic of the tumor microenvironment. Far from being a disadvantage, hypoxia is an undeniable force for the tumor cell. Functional benefits of hypoxia include epigenetic modifications, tumor vascularization, modified metabolism, signaling of metastasis, invasion and extravasation, cancer stemness, and innate immune activation, all of which are under the control of complex molecular pathways driven by the transcription factor the hypoxia-inducible factor (HIF) (3, 4). These changes help cells to proliferate and resist cell death induced by chemotherapy or radiation. Among the processes activated during tumor growth, metabolism, and more specifically glycolysis, is the one that is the most exacerbated by hypoxia (5, 6). To counterbalance mitochondrial generation of ROS that interfere with cell survival, HIF-1 activates pyruvate dehydrogenase kinase (PDK1) to block the conversion of pyruvate to acetyl CoA resulting in decreased flux through the tricarboxylic acid (TCA) cycle (7, 8). Moreover, HIF-1 upregulates the expression of *COX4-2*, present in complex IV, and the mitochondrial protease *LONP1*, which in turn degrades COX4-1. COX4-2 is then more efficient at facilitating electron transfer to O_2_ and thereby protects the cell from oxidative damage in hypoxia (9). HIF-1-mediated inhibition of MYC (10) and PGC-1 also results in reduced mitochondrial biogenesis (11). However, cancer cells have selectively found new mechanisms that promote their survival.

In this mini review, we highlight a new hypoxic mechanism that modulates the amount (decrease) and structure (a cleaved form lacking its C-terminus) of the most abundant protein of the outer mitochondrial membrane (12), the voltage-dependent anion channel (VDAC), which profoundly impacts cancer cell proliferation and survival.

## The Voltage-Dependent Anion Channel: Function, Organization, and Structure

Voltage-dependent anion channel plays a key role in both mitochondrial metabolism and cell death, acting as a convergent point of control (13, 14). The function of VDACs as a pore seems quite clear-cut, but other suspected more complex functions are yet to be elucidated. VDACs play a crucial role as a gatekeeper for the entry and exit of many metabolites. They mediate and gate the flux of small ions (Cl^−^, K^+^, Na^+^) with a preference for anions and metabolites (NADH, citrate, succinate, glutamate, pyruvate, and glucose) and act as channels that constitute the main pathway for passage of ATP/ADP (15–18). In addition to their role in bioenergetics, VDACs act as a scaffold through interactions with numerous proteins. They are anchors for pro- and anti-apoptotic proteins, respectively, of the hexokinase (HK) (19, 20) and Bcl-2 families (21–24) of proteins, which contribute to the balance between survival and cell death.

Voltage-dependent anion channels exist as three isoforms: VDAC1, VDAC2, and VDAC3, encoded by three different genes. The three human *VDAC* genes share the same number of exons for each gene isoform (25). The human *VDAC1* gene spans about 30 kb localized on the chromosome 5q31-32 (26), the human *VDAC2* has been mapped to chromosome 10q22 and is 16.4 kb in length whereas the human *VDAC3* is localized on chromosome 8p11.21 with a length of 14.3 kb. The *VDAC2* gene uses several polyadenylation sites, thus giving rise to multiple mRNA, whereas the *VDAC3* gene presents an alternative splicing event that corresponds to an additional ATG. At the protein level and in mammals, VDACs share ~70% identity with a very similar molecular mass of 30–35 kDa. They are known to be expressed ubiquitously in mammalian mitochondria, where VDAC1 remains the most abundantly expressed of the three isoforms (27). VDAC1 has also been detected in the plasma membrane of human lymphocytes (27, 28) and in the sarcoplasmic reticulum (29).

Analysis of the structure of VDACs revealed a 19-stranded β-barrel fold (13), yet only 13 of these strands form the wall of the channel. The N-terminal region of VDACs is very dynamic and exposed to the cytoplasm but located inside the pore. It acts as the voltage sensor and maintains the channel in an open or closed status (25). In an open-state configuration, VDACs are capable of passing millions of ATP molecules per second *in vitro* (17) and up to 100,000 ATP molecules per second under physiological conditions (16, 17), using at least five different trajectories (30). By contrast, very little is known about the function of the C-terminus of VDACs. It possesses NAD^+^-binding sites, considered essential for glycolysis (31). Finally, VDAC1 can oligomerize and assemble into a dynamic equilibrium of dimers, trimers, tetramers, and higher oligomers (32). These conformational changes could occur upon induction of apoptosis (33). However, the function of VDAC1 oligomers is not known. They may contribute to the stabilization of the protein (34) and may offer a more stable platform to anchor HKs I and II (32).

## The Voltage-Dependent Anion Channel: Modifications, Silencing, and Over-Expression

**Figure 1:**
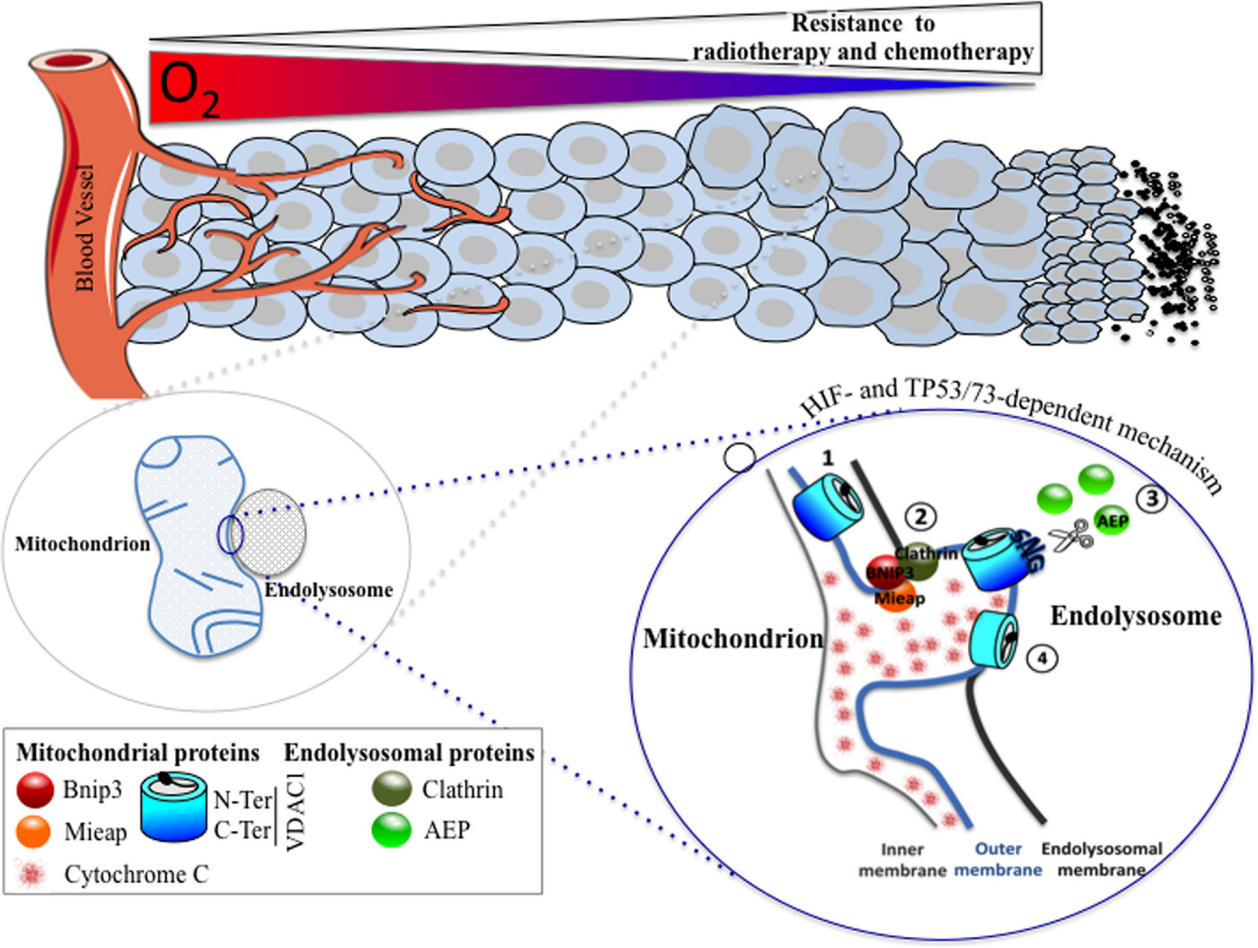
**A HIF- and TP53/73-dependent model that potentiates tumor cell survival in hypoxia through the formation of enlarged mitochondria that interact with endolysosomes to modify mitochondrial VDAC1, an ATP channel-regulating metabolism and apoptosis**. As oxygen diffuses from a vessel, a decreasing gradient in the oxygen concentration occurs in the adjacent tissue (top). As the level in oxygen decreases, resistance to radiotherapy and chemotherapy increases since the former requires oxygen for DNA damage, and the latter depends on the limits of tissue diffusion of the drug. Hypoxic cells with resistance to chemotherapy show the presence of enlarged hypoxic mitochondria (magnified on the bottom left). Microfusion between the mitochondrial outer membrane and an endolysosomal membrane takes place (magnified on the bottom right), and a cleaved form of VDAC1 is produced, according to the following steps: (1) VDAC1 is exposed to an endolysosome, (2) a complex formed of BNIP3/Mieap (mitochondrial proteins) and clathrin (endolysosomal protein) maintained microfusion of both membranes, (3) the endolysosomal asparagine endopeptidase (AEP), in contact with the mitochondrial outer membrane, specifically cleaves VDAC1, and (4) VDAC1-ΔC promotes resistance to apoptosis and a blockade in cytochrome *c* release.

Post-translational modifications, changes in expression, or even mutation in VDACs profoundly disrupt metabolism and, thus, the balance between cell survival and cell death. The three isoforms of VDAC can be post-translationally modified by phosphorylation and acetylation at multiple sites (35). The role of VDAC1 phosphorylation remains unclear, as it is difficult to study these modifications on highly hydrophobic integral mitochondrial outer membrane proteins. The impact of these modifications has been studied mostly in the context of apoptosis. However, no direct relationship to VDAC function or activity has been demonstrated. The relevance of acetylation remains to be determined. Recently, our studies showed a new form of post-translational modification of VDAC1; C-terminal truncation of the protein to give VDAC1-ΔC (discussed in Section “The Hypoxic Mitochondrial Phenotype and VDAC1-ΔC”) (Figure 1). This modification occurred specifically under hypoxic conditions. This hypoxic form was associated in some cancer cell lines with resistance to chemotherapy-induced apoptosis, a higher output of ATP and was found in late stage tumors of patients with lung cancer (36). A mutation in VDAC1 that resulted in the removal of 60% of the length of the C-terminal region has been described in colorectal and gastric cancers, but the consequence on metabolism and apoptosis is still to be determined (37).

Deletion of genes coding *Vdac* in mice models has provided information concerning the functions of VDACs. *Vdac1* and *Vdac3* heterozygote embryonic stem cells have been generated to obtain hetero- and homozygote knockout mice (38, 39). *Vdac1*^−/−^ mice did not meet the normal Mendelian pattern, suggesting partial embryonic lethality at days 10.5–11.5. For the mice that survived, multiple respiratory defects appeared in skeletal and cardiac muscles. The mice were fertile but were retarded in growth. Mitochondria from muscle fibers of skin contained enlarged mitochondria with very compact cristea. *Vdac3*^−/−^ mice survived with defects in mitochondrial respiration in heart tissue. Males were also infertile with alterations to their sperm. A combination of both *Vdac1*^−/−^ and *Vdac3*^−/−^ deletions was not lethal. Finally, the homozygote mice lacking *Vdac2* died during development (40). Although it seems surprising not to find VDACs in a tissue, we recently reported, using the Cancer Genome Atlas (TCGA) data sets from 89 cancer studies, that deletion of the *VDAC1* gene is found in some cancer types (41). Even if the *VDAC1* gene was mainly heterologously lost, some homologous loss was found. Clear cell renal cell carcinoma (ccRCC) and ovarian cancers seemed to be the most affected by the homologous loss of the *VDAC1* gene, whereas the heterologous loss was almost ubiquitous. It might be interesting to further examine these data to check if the *VDAC2* and *VDAC3* genes are also deleted or if compensation has taken place to counterbalance for the homologous loss of *VDAC1*. New aspects of the function of VDAC1 were highlighted in our recent transcriptome analysis of mouse embryonic fibroblasts (MEFs) knocked out for *Vdac1* (41). We characterized the cellular and molecular phenotype of both *Vdac1*^−/−^ MEF and MEFs transformed with the pBabe-RAS^v12^ vector, *Vdac1*^−/−^ RAS MEF. Our results pointed to alterations in programs controlling HIF-1, cell death, and survival, as well as cell proliferation and motility. We confirmed the presence of alterations in OXPHOS and glycolysis in knocked out cells, which was accompanied by a higher level of apoptosis. Of note, *Vdac1*^−/−^ MEF and *Vdac1*^−/−^ RAS MEF grew better in hypoxia, by maintaining respiration and promoting glycolysis. *Vdac1*^−/−^ RAS MEF formed tumors faster than Wt RAS MEF in nod-scid mice. Moreover, after dissection of the various mechanisms involved, our results showed a strong impact of VDAC1 on tumor development, through alterations in the inflammatory response as a result of an abnormal vasculature due to ROS production and HIF-1α stabilization. Changes in metabolism were also observed in both *Vdac1*^−/−^ MEF and *Vdac1*^−/−^ RAS MEF. The first surprise came from the impact of hypoxia on the behavior of *Vdac1*^−/−^ cells, in particular on cell proliferation. These initial results lead us to revisit the Mendelian ratio observed in the *Vdac1*^−/−^ mice. Indeed, a large number of *Vdac1*^−/−^ embryos did not survive (60%), whereas the *Vdac1*^−/−^ embryos that survived gave an almost normal phenotype. Is it possible that the non-viable *Vdac1*^−/−^ embryos were not exposed to low oxygen concentrations during development and, therefore, apoptosis prevailed? Our second hypothesis is that non-viable *Vdac1*^−/−^ embryos may have developed dysfunctional blood vessels during embryogenesis. The second surprise came from our result that suggested the involvement of VDAC1 in modulating the structure of blood vessels and in enhancing the inflammatory response (41). Indeed, accumulation of ROS in *Vdac1*^−^*^/^*^−^ RAS MEF-derived tumors triggered HIF-1α stabilization, abnormal vasculature, and leakage of red blood cells, thus generating an inflammatory response that resulted on a strong impact on VDAC1 tumor development. To our knowledge, this is the first time that VDAC1 has been connected to such events. These pathways should not be neglected in the future when considering VDAC1 as a therapeutic target.

Exogenous over-expression of VDAC1 in different cell lines always seems to be linked to apoptosis (24, 25, 42, 43). However, the impact of VDAC1 directly or indirectly on apoptosis is not yet clear. It may depolarize the inner membrane (44) as it could trigger the mitochondrial permeability transition pore (MPTP) (45). However, we showed that VDAC1 was over-expressed in lung adenocarcinomas tumor tissue from 44 patients (36). More recently, after analysis of the same TCGA data sets, as described above, expression of *Vdac1* was also gained and amplified (41). The *in vitro* results contrast with the *in vivo* ones. It is easy to hypothesize that as VDAC1 regulates metabolism through its association with HK, cancer cells draw a substantial profit from increasing glycolysis. Similarly, because of its association with members of the Bcl-2 family, cancer cells, again, take advantage of such an association by minimizing apoptosis.

## The Hypoxic Mitochondrial Phenotype and VDAC1-ΔC

Mitochondria are dynamic organelles that undergo membrane remodeling through cycles of fusion and fission (Figure 1). This balance controls the mitochondrial structure and, thus, mitochondrial activity. The key factors regulating fusion are the dynamin-related GTPases mitofusin 1 (Mfn1) and 2 (Mfn2), and optic atrophy 1 (OPA1) that mediate the OMM, while the dynamin-related protein 1 (DRP1) regulates the opposing process of fission of the inner mitochondrial membrane (IMM) (46). Cells lacking mitochondrial fusion show changes in mitochondrial shape associated with a loss of their membrane potential, a reduced growth rate, and a lower activity of respiratory complexes (47). By contrast, forced expression of Mfns resulted in the formation of clusters with enlarged mitochondria due to the fusion of the OMM (48). In 2010, we reported that a number of human cancer cells, including colon carcinoma cells (LS174) and non-neoplastic CCL39 lung fibroblasts exposed to long-term hypoxia (72 h – 1% O_2_) showed a change in mitochondrial phenotype from a tubular network to an enlarged morphology (49). The modification of the shape of mitochondria observed in LS174 cells was HIF-1-dependent (36). The formation of these enlarged mitochondria resulted from hyperfusion as expression of Mfn1 was increased in hypoxia and as silencing of the expression of Mfn1 reverted the mitochondrial morphology. Moreover, we reported that Bcl-2/adenovirus E1B 19-kDa interacting protein (BNIP3) and BNIP3 like (BNIP3L), two pro-autophagy proteins from the Bcl-2 family, also participated in the dynamic process of fusion induced in hypoxia. Finally, cytochrome *c* was retained inside these structurally unusual mitochondria when the cells were treated with staurosporine, a pro-apoptotic drug. We concluded that cells with enlarged mitochondria were more resistant to cell death than normoxic cells and that these cells possessed a selective growth advantage.

We were, therefore, faced with the challenge of exploring the underlying mechanisms that lead to the protective phenotype of hypoxic cancer cell. We found that VDAC1 was detected in hypoxia as a smaller than usual form (26 kDa rather than 30 kDa on SDS-PAGE). The amount of the 30 kDa form was decreased by around 50% with a parallel increase in the smaller 26 kDa form in hypoxia. Using a VDAC1 antibody directed to the C-terminus of VDAC1 that did not detect this fast migrating form of VDAC1 on immunoblots (36) and after analysis by mass spectrometry (50), we concluded that it was a form truncated in the C-terminal region: VDAC1-ΔC. After reconstitution into a planar lipid bilayer system, VDAC1-ΔC presented a similar but not identical channel activity and voltage dependency as VDAC1. At a higher voltage of −40 mV, the full-length channel showed two major conducting states with higher occupancy at the closed substate, whereas VDAC1-ΔC showed a higher open-state occupancy in comparison to the occupancy of low-conducting substates. Moreover, at the high voltages, VDAC1-ΔC showed a slightly higher conductance than VDAC1 (36). Indeed, we observed an increase in ATP levels in cells in hypoxia when VDAC1-ΔC and enlarged mitochondria were present. We also demonstrated that VDAC1-ΔC associated with the same partners as VDAC1, i.e., HKI/II and Bcl-X_L_. Anchoring of HKI/II is probably involved in the exacerbated metabolism of tumor cells in hypoxia in the presence of VDAC1-ΔC. Both the association of VDAC1-ΔC with HK and the increase in the expression of HKI/II in hypoxia increased OXPHOS and glycolysis. Thus, VDAC1-ΔC seems to control cell survival in hypoxia by regulating the export of ATP and probably NADH and brings advantage to cancer cells in promoting survival *via* mitochondria that are probably not as dormant as previously described. We do not know yet whether the conformation and the oligomerization of VDAC1-ΔC are different to that of VDAC1.

The VDAC1–HK complex has been reported to also play an important role in apoptosis (14). Thus, the VDAC1–HK complex already represents a target for cancer therapy, using, for example, specific VDAC1-based peptides that disrupt the connections between these proteins (51). HK provides an apoptosis-suppressive capacity by interfering with the ability of Bax to bind to mitochondria and induce apoptosis (52, 53). In addition, Bcl-X_L_, which interacts with VDAC1-ΔC (36), was found to protect cells from apoptosis via a block in the Bax–Bak interaction, subsequently preventing cytochrome *c* release (54, 55). Currently, cytochrome *c* is considered to play an important role in the resistance to cell death observed in cells with enlarged mitochondria and VDAC1-ΔC. We have previously shown that resistance to chemotherapy was linked to the phenotype of enlarged mitochondria. Subsequently, we demonstrated that silencing of VDAC1/VDAC1-ΔC in hypoxia or re-exposure of cells to normoxia, which inhibited the formation of VDAC1-ΔC, restored the sensitivity of the cells to apoptosis. We also showed that the mitochondrial transmembrane potential was unchanged in hypoxia and that cytochrome *c* was not released in the presence of staurosporine-induced apoptosis, whereas the transmembrane potential was decreased in normoxic cells under these conditions. We showed that cytochrome *c* was trapped inside the mitochondrial intermembrane space due to a change in the mitochondrial conformation and a hypothetical modification to VDAC oligomerization. These interactions occur specifically in hypoxia, when cancer cells are known to be highly resistant to anti-cancer treatments, and may, therefore, be exploited for therapy in the future.

Finally, we investigated further the mechanism behind the hypoxic regulation of the truncated form of VDAC1. We found that the cleavage of VDAC1 was dependent on TP53 or p73 as only cells expressing p53 (LS174, A549, or HepG2 cells) or p73 (HeLa cells) contained the hypoxic VDAC1-ΔC. Moreover, silencing of *TP53* and/or *HIF-1α* diminished VDAC1 truncation and, thus, cell survival in the presence of staurosporine. Mieap, a TP53-inducible protein that controls mitochondrial quality was also involved, as silencing of *MIEAP* diminished also the hypoxic VDAC1-ΔC. We found that bafilomycin A1 and chloroquine, two compounds that increase the lysosomal pH, inhibited the cleavage of VDAC1 to VDAC1-ΔC, suggesting implication of lysosomes. This was confirmed by electron microscopy, which showed microfusion between mitochondria and endolysosomes and by cleavage of VDAC1 by an endolysosomal asparagine endopeptidase (AEP). Analysis by mass spectrometry of VDAC1-ΔC showed cleavage at asparagine 214 and glycine 213. Of note, AEP is also regulated by TP53 (56). Moreover, we reported that BNIP3, already known to be involved in regulating mitochondria morphology in hypoxia (36), acted as a docking site for lysosomes together with clathrin, a protein involved in multiple membrane vesicle trafficking pathways (57). This mechanism was identified not only *in vitro* but also *in vivo* in patients with lung cancer. We propose that this novel mechanism is a readout of mitochondrial–endolysosomal microfusion in hypoxia, *in vitro* and most importantly *in vivo*, and represents an additional defense mechanism that cancer cells have developed to resist chemotherapy.

## Targeting VDAC1 for Therapy

Studies into cellular metabolism have lead to the characterization of a number of drugs that have already showed promise in pre-clinical and clinical trials. However, the quest for new therapeutic targets is hampered by the fact that cells show a great degree of plasticity, which already augurs the challenges we face. Studying metabolism *per se* is important and will allow us to identify new targets. However, metabolism should be studied in the context of a changing microenvironment (hypoxia, pH, changes in concentrations of metabolites, etc.) and in the context of malignant transformation. Thus, VDAC1-ΔC appears to be an interesting therapeutic target. Various compounds have already been identified for their capability to directly interact with and modify the activity of VDAC. Avicins (closes VDAC) (58), acrolein (used in Alzheimer’s disease to carbonylate VDAC) (59), erastine (binds to VDAC2) (60), endostatin (inducing PTP opening) (61), fluoxetine and cisplatin (inhibition of PTP opening and apoptosis) (62, 63), furanonaphthoquinones (induces VDAC-dependent apoptosis) (64), and oblimersen (blocks channel activity) (65, 66) are chemicals that will be tested under hypoxic conditions in the near future. In addition, VDAC-based peptides, novel pro-apoptotic agents that specifically target domains for interaction with HK, Bcl-2, and Bcl-X_L_, could be an interesting alternative to chemicals to restore the sensitivity to apoptosis in hypoxia (67).

We hope that, in the near future, this hypoxia/VDAC1-ΔC duo will meet the expectations that we have discussed in this review.

## Author Contributions

The author confirms being the sole contributor of this work and approved it for publication.

## Conflict of Interest Statement

The author declares that the research was conducted in the absence of any commercial or financial relationships that could be construed as a potential conflict of interest.
